# Extra-gastrointestinal Stromal Tumor of the Urinary Bladder: A Report of a Rare Case and Literature Review

**DOI:** 10.7759/cureus.84435

**Published:** 2025-05-19

**Authors:** Swapnil S Kushwaha, Abhilash Goyal, Shikha Goja

**Affiliations:** 1 Urology, Max Super Speciality Hospital, Saket, Delhi, IND; 2 General Surgery, Vardhman Mahavir Medical College and Safdarjung Hospital, Delhi, IND; 3 General and Minimal Access Surgery, All India Institute of Medical Sciences, Guwahati, Guwahati, IND; 4 Cardiothoracic and Vascular Surgery (CTVS), All India Institute of Medical Sciences, New Delhi, New Delhi, IND

**Keywords:** cd117 expression, extraintestinal gist, gross hematuria, partial cystectomy, urinary bladder tumor

## Abstract

Extra-gastrointestinal stromal tumors (eGISTs) are rare tumors that occur outside the gastrointestinal tract but share similar histological and molecular characteristics as GISTs. This report highlights an exceptionally rare case of eGISTs in the urinary bladder of a 40-year-old man who presented with painless gross total hematuria. Computed tomography (CT) revealed a solitary bladder mass, approximately 3 × 3 cm over the anterior wall, with extravesical extension into the urachal remnants. He was diagnosed with eGIST of the urinary bladder based on histopathology and immunohistochemistry after cystoscopy and transurethral resection. The patient underwent open partial cystectomy and excision of the urachus, with an uneventful recovery and no recurrence at 28 months follow-up. This case report underscores the importance of recognizing and appropriately managing bladder eGISTs.

## Introduction

Gastrointestinal stromal tumors (GISTs) are mesenchymal tumors found in the gastrointestinal (GI) tract, with an incidence of 10-15 cases per million worldwide. GISTs most often occur in individuals aged 60-70 years, with no gender preference. Symptoms arise either from a mass effect, causing early satiety or obstruction, or from ulceration and tumor rupture, resulting in pain, gastrointestinal bleeding, anemia, or perforation. They are typically characterized by the expression of markers such as c-kit (CD117), CD34, and DOG-1. Additionally, approximately 30%-50% of these tumors demonstrate positivity for alpha-smooth muscle actin while consistently being negative for S100 and desmin [1,2]. A small subset of these tumors, known as extra-gastrointestinal stromal tumors (eGISTs), arises outside the GI tract. These account for about 5%-10% of all GISTs and can be found in diverse locations, including the omentum, retroperitoneum, mesentery, pancreas, gall bladder, liver, spleen, urinary bladder, and prostate [3]. Despite their atypical location, eGISTs retain the same histological, immunohistochemical, and molecular genetic characteristics as their gastrointestinal counterparts [1,4]. Bladder involvement by eGISTs is extremely rare, with only a few cases documented in the literature [4-9]. This article presents a rare case of bladder eGIST, discussing its management and reviewing relevant literature to provide a comprehensive overview of this rare tumor.

## Case presentation

A 40-year-old man presented with three episodes of painless visible hematuria, associated with clots in the last five days. There were no lower urinary tract symptoms and associated systemic symptoms, such as fever, fatigue, or weight loss. His medical history was unremarkable, and he was not on any drugs or anticoagulants. On physical examination, the patient appeared pale, but his vital signs were stable. There were no detectable abnormalities upon abdominal examination. The important laboratory test results are indicated in Table 1.

**Table 1:** Key laboratory parameters

Parameter	Value	Reference range
Hemoglobin	7.6 g/dL	12-16 g/dL
Blood urea	17 mg/dL	7-20 mg/dL
Serum creatinine	1.03 mg/dL	0.7-1.2 mg/dL

**Figure 1:**
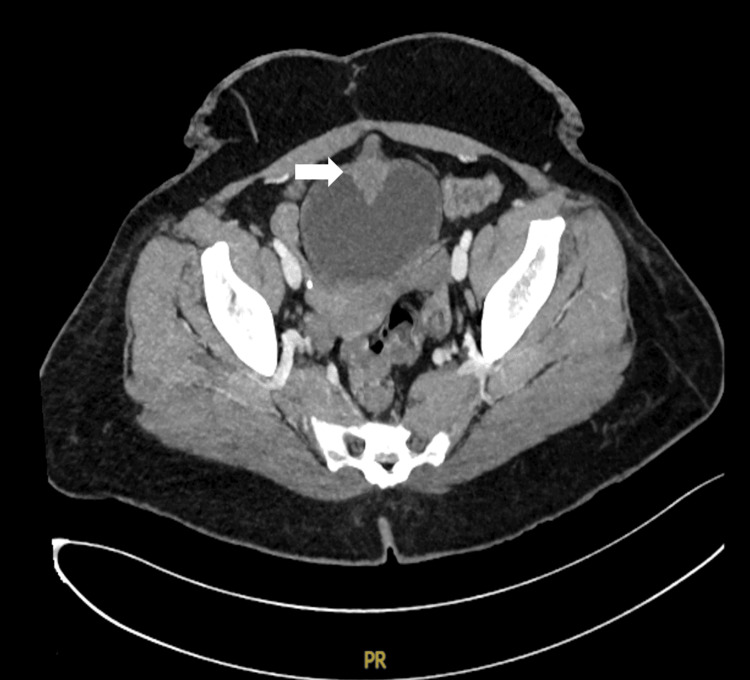
CT axial section demonstrating soft tissue density over the anterior wall of the urinary bladder White arrow pointing to the tumor CT: computed tomography

**Figure 2:**
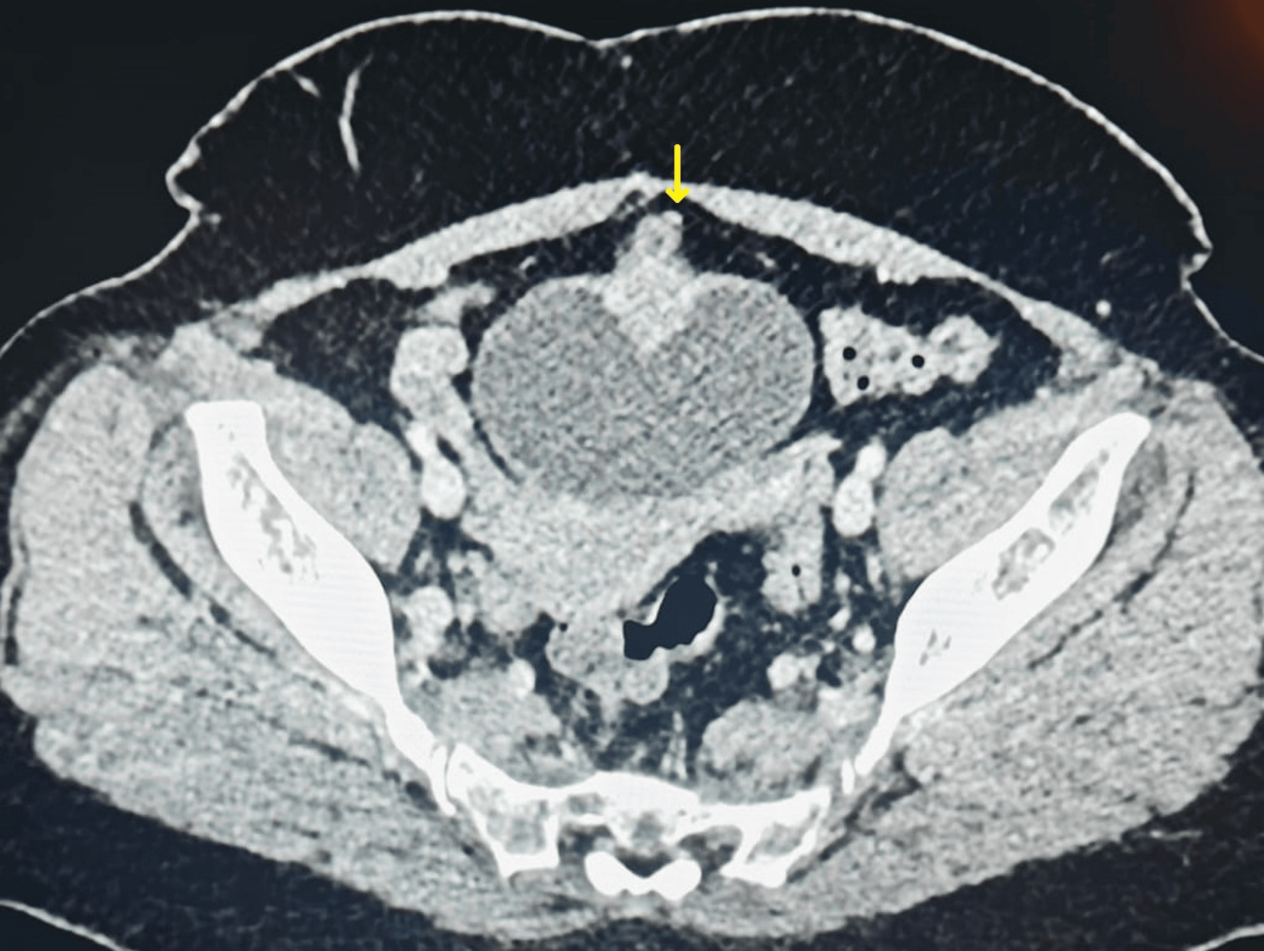
CT axial section showing extravesical extension into urachal remnants Yellow arrow pointing toward the extravesical extension CT: computed tomography

A contrast-enhanced computed tomography (CT) scan of the abdomen and pelvis revealed a solitary irregular homogenously enhancing soft tissue mass, measuring approximately 3 × 3 cm, located on the anterior wall of the urinary bladder, with extension into urachal remnants (Figure 1 and Figure 2).

**Figure 3:**
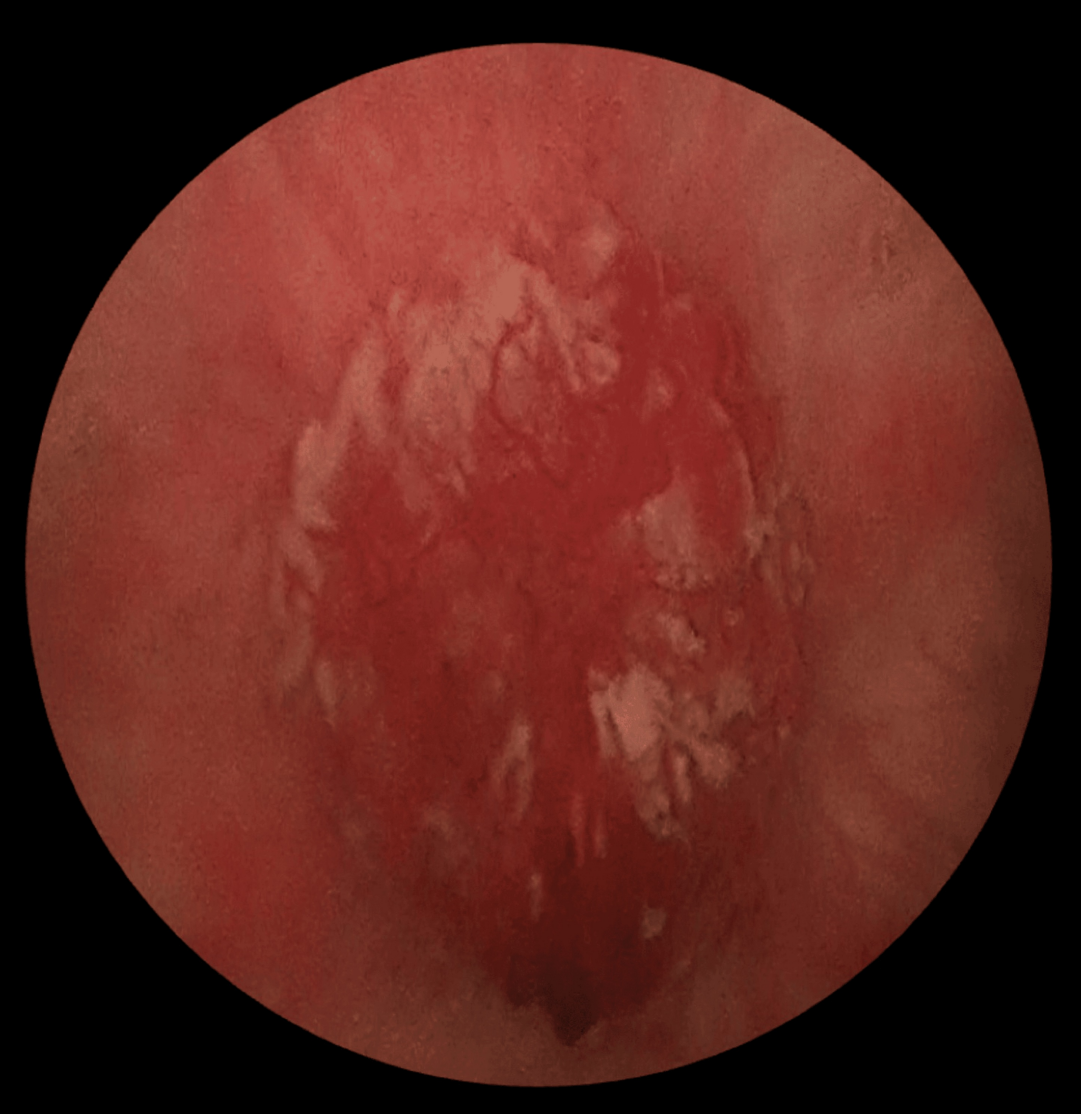
Tumor at the anterior wall of the urinary bladder on cystoscopy

**Figure 4:**
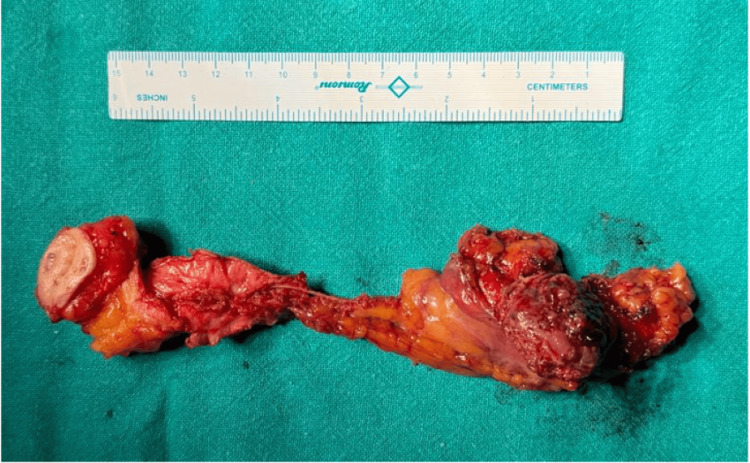
Completely resected specimen of the tumor bed, urachal remnant, and umbilicus

Given these findings, the patient underwent cystoscopy and transurethral resection, which revealed a round-shaped tumor with a broad base over the anterior wall of the urinary bladder of size 3 × 3 cm (Figure 3), with adequate bladder capacity. Gross complete resection of the bladder tumor was performed. The remainder of the bladder appeared normal, with no additional lesions. Histopathological analysis of the resected tissue showed that the tumor was composed of uniform spindle cells with abundant acidophilic cytoplasm, arranged in interwoven bundles or swirls, with minimal atypia (<5 mitoses per 50 high-power field (HPF)). Immunohistochemical staining revealed that the tumor cells were strongly and diffusely positive for CD117 and smooth muscle actin, while negative immunohistochemical staining results were obtained for CD34, desmin, and S100, leading to a diagnosis of extra-gastrointestinal stromal tumor (eGIST). Given the tumor's extravesical extension into the urachal remnants, the decision was made to perform an open partial cystectomy with excision of the urachal remnants and the umbilicus (Figure 4).

**Figure 5:**
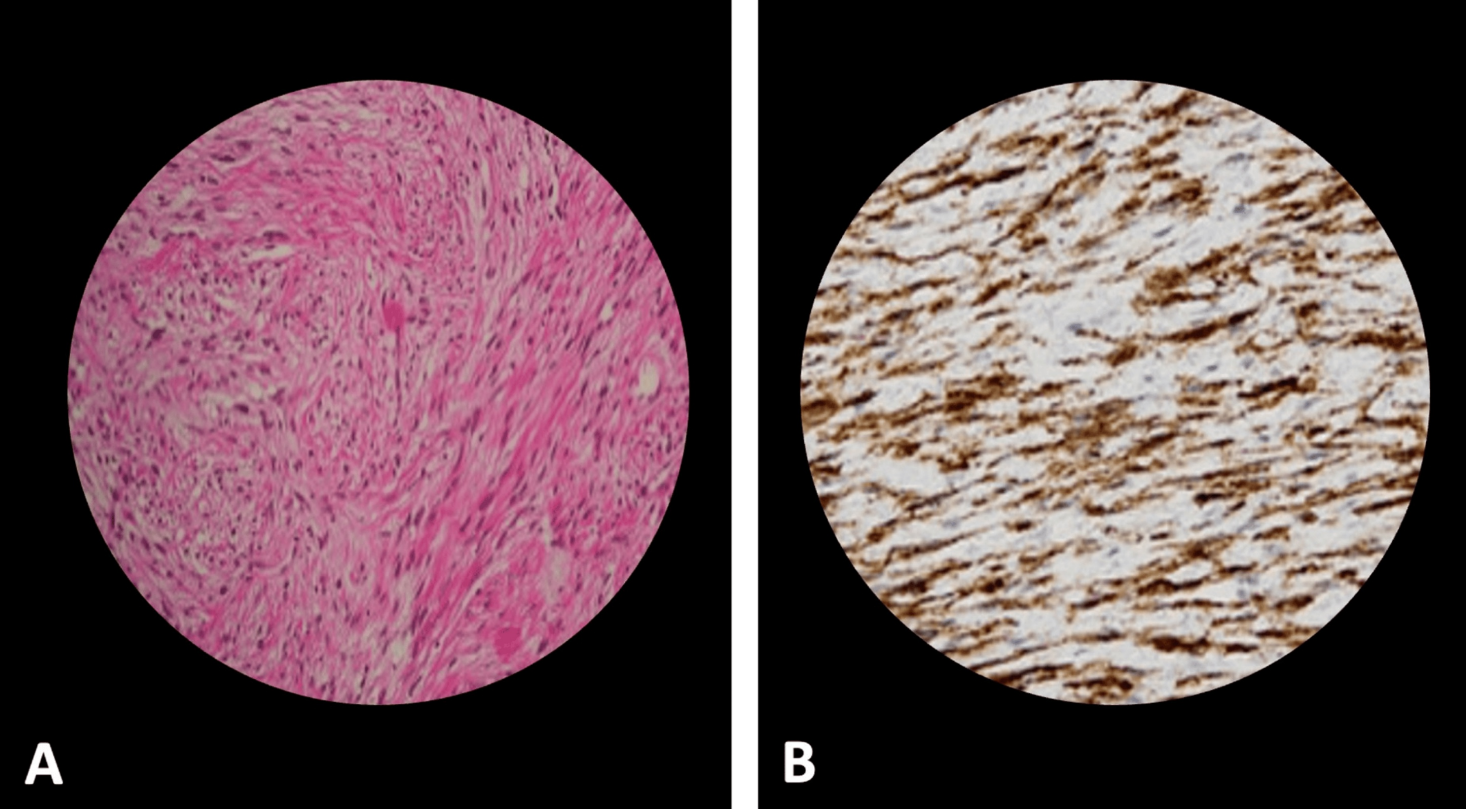
A: Spindle cells arranged in interwoven bundles or swirls with little atypia, B: tumor cells showing strong and diffuse positivity for CD117 on immunohistochemical staining

The surgery was completed in 240 minutes with a blood loss of 300 mL, and a perivesical drain and per urethral catheter were placed. The patient's postoperative course was uneventful, and he was discharged on the seventh day after surgery, and the catheter was removed on the 10th day. Follow-up histopathological examination confirmed the initial diagnosis of eGIST based on the immunoreactivity for CD117 (Figure 5). Postoperatively, the patient did not receive adjuvant molecular-targeted therapy because of complete resection, small tumor size, and minimal atypia on microscopy. At 28 months follow-up, the patient remained asymptomatic, with no evidence of disease recurrence on six-monthly cystoscopy and CT scan of the abdomen and pelvis.

## Discussion

GISTs are mesenchymal tumors, albeit uncommon, and are most frequent in the GI tract. They may show up in any part of the GI tract. However, the stomach (50%-60%) is the most common site of occurrence, followed by the small intestine (25%). Their location outside the gastrointestinal tract is quite rare, and it is thought to be preponderantly in the mesentery, omentum, or retroperitoneum; therefore, they are called registers. The urinary bladder is an extremely uncommon site for eGISTs [3]. As far as we could possibly know, following a literature review for eGISTs, we found only a few patients with bladder eGISTs [4-9].

GISTs are commonly characterized by the expression of c-KIT protein (CD117) and CD34, with 30%-50% exhibiting focal positivity for smooth muscle actin. All GISTs are negative for S11 and desmin [10]. This immunohistochemical profile is instrumental in distinguishing GISTs from leiomyomas and schwannomas. Recently, mutations in exon 11 of the *c-kit* gene have been identified as a new molecular genetic marker for a specific subset of GISTs. The expression of c-KIT protein in immunohistochemistry has been characterized as the most significant component for diagnosing GISTs and, therefore, helps in the diagnosis of tumors, particularly in distinct sites such as the prostate gland and bladder [11].

The pathogenesis of GISTs is not yet fully unfolded. Some hypothesized it to originate from interstitial cells of Cajal (ICC), the pacemaker cells that surround the nerve plexus of the gastrointestinal tract. The occurrence of GIST-like tumors in pelvic locations, such as the urinary bladder or prostate, suggests that eGISTs may originate from primitive mesenchymal precursor cells rather than from ICC. The hypothesis of GISTs arising from mesenchymal precursor cells also provides a more coherent explanation for their presence in the omentum, mesentery, and retroperitoneum, independent of the tubular gastrointestinal tract [8]. However, some continue to believe it originates from ICC present in the urinary bladder also [9].

eGISTs have non-specific clinical manifestations based on the site of occurrence. eGISTs of the urinary bladder commonly present with gross hematuria, often resulting from bleeding due to tumor infiltration of the mucosal layer. Additionally, imaging techniques such as ultrasound, CT, and magnetic resonance imaging (MRI) lack definitive diagnostic criteria for eGISTs. The current diagnostic approach for eGISTs relies on immunohistochemical analysis, typically focusing on the expression of c-KIT. In cases where the tumor morphology suggests an eGIST, but c-KIT expression is absent, the use of the DOG-1 marker is recommended to establish the diagnosis [12].

For eGISTs, surgical resection is currently the preferred treatment option, as they are considered to be refractory to conventional chemotherapy and radiotherapy. Complete surgical resection with negative microscopic margins is the most effective form of treatment for primary localized GISTs [13]. As the molecular mechanisms of GIST were not fully understood till two decades ago, there was no effective medical therapy for unresectable or metastatic GIST. The seminal discovery of the expression of CD117 in GIST was a major breakthrough. In more than 80% of GIST, *c-kit* proto-oncogenes have gain-of-function mutations that cause constitutive activation of c-KIT protein, a receptor tyrosine kinase (RTK), leading to tumorigenesis and proliferation [14-16]. The c-KIT receptor tyrosine kinase inhibitor (TKI), imatinib mesylate, is currently being used for treating unresectable or metastatic eGISTs, based on the importance of the *c-kit* gene in the development of GISTs.

About 50% of patients who had a curative complete surgical resection developed recurrence on subsequent follow-up, even after several years [2]. The factors implicated in the progression and aggressive behavior of tumors have been debated for years. Several risk classifications are known now, which include tumor size and location, mitotic index, and presence of tumor rupture [1]. To improve the outcomes and prognosis of patients who are at a high risk of recurrence despite curative resection, numerous retrospective and prospective studies have been done to evaluate the efficacy of imatinib as adjuvant therapy, based on the high response rate of this agent in unresectable or metastatic GIST. Recently, a nomogram was developed and validated by Gold et al. to select patients for adjuvant TKI based on a prediction of the risk of recurrence after surgery [17]. National Comprehensive Cancer Network (NCCN) and European Society for Medical Oncology (ESMO) guidelines both state that imatinib can be considered in adjuvant settings as a treatment option for patients at high risk of recurrence [18]. The daily dose of imatinib is 400 mg for a duration of at least three years, and it is recommended for high-risk GIST patients.

Recent literature suggests that imatinib can be considered in neoadjuvant settings if surgical comorbidity can be reduced by downstaging the tumor. Neoadjuvant therapy is expected to achieve organ-preserving surgery, avoid rupture of tumors, and reduce complications. Additionally, imatinib, used as neoadjuvant therapy, has been shown to reduce tumor size, allowing for a more conservative and less invasive R0 resection, especially for gastrointestinal stromal tumors (GISTs) located in the duodenum and rectum. Currently, NCCN guidelines state that for multi-visceral resection in primary GIST, imatinib may be beneficial as neoadjuvant therapy [19]. Similarly, the ESMO recommends neoadjuvant imatinib in patients with localized disease if it results in less extensive surgery and a lower risk of tumor rupture and bleeding [19]. Neoadjuvant therapy typically involves a daily dose of 400 mg; however, for cases with a KIT exon 9 mutation, an increased dose of 800 mg is recommended. The optimal duration is controversial, but most agreed that at least 3-6 months of therapy may be required before surgery. Response evaluation is performed using positron emission tomography (PET) scans, which can provide an indication of imatinib activity within 2-4 weeks of therapy. There is currently no universally agreed-upon approach for adjuvant therapy following surgery in patients who have undergone neoadjuvant therapy. Only imatinib is evaluated for GISTs, and there are no comparative trials on whether sunitinib or regorafenib is effective in neoadjuvant or adjuvant settings. However, in EGISTs, the use of imatinib is limited in clinical experience, owing to the rarity of the condition, and needs further support from more clinical cases in the future.

GIST patients are usually monitored through CT scans with contrast or MRI, particularly for younger individuals, to minimize radiation exposure. NCCN guidelines suggest abdominal/pelvic CT with contrast every 3-6 months for 3-5 years after GIST surgery and then annually, except for small tumors under 2 cm, which may require less frequent surveillance. According to ESMO guidelines, risk assessment can guide follow-up, with high-risk patients undergoing CT or MRI every 3-6 months for three years during adjuvant therapy, three months after, and annually for five more years. Low-risk GIST patients may have scans every 6-12 months for five years, but very low-risk tumors do not have specific follow-up recommendations [20].

## Conclusions

Although eGISTs of the urinary bladder are exceptionally rare, the urologist must remain aware of their possibility and be prepared to manage them effectively, if diagnosed. The clinical presentation of these tumors can closely mimic that of urothelial carcinoma, often leading to diagnostic challenges. As such, definitive diagnosis relies heavily on the combination of cystoscopic examination, histopathological evaluation, and immunohistochemical staining, which collectively help distinguish eGISTs from other bladder malignancies. Surgical resection remains the cornerstone of treatment for eGISTs, with the goal of achieving complete tumor excision, complemented by molecular-targeted therapy, particularly in high-risk cases, to reduce the likelihood of relapse following surgery. Long-term, regular follow-up is essential for monitoring patients postoperatively.
